# Triclosan-coated sutures to reduce surgical site infection in abdominal gastrointestinal surgery: A meta-analysis and systematic review

**DOI:** 10.1016/j.sopen.2023.09.009

**Published:** 2023-09-14

**Authors:** Norikatsu Miyoshi, Shiki Fujino, Clinical Study Group of Osaka University, Colorectal Cancer Treatment Group (CSGOCG)

**Affiliations:** a Department of Gastroenterological Surgery, Osaka University Graduate School of Medicine, Osaka, Japan; b Department of Innovative Oncology Research and Regenerative Medicine, Osaka International Cancer Institute, Osaka, Japan; c Central Clinical School, Monash University, Melbourne, Australia

**Keywords:** Triclosan-coated sutures, Surgical site infections, Gastrointestinal surgery, meta-analysis

## Abstract

**Background:**

Previous randomized trials evaluated the effectiveness of triclosan-coated sutures for fascial closure in preventing surgical site infection (SSI). However, available evidence remains still inconclusive. We aimed to evaluate the effectiveness of triclosan-coated sutures in fascia closure in preventing postoperative SSI in elective gastrointestinal surgery.

**Meta-analysis:**

A meta-analysis included present outcomes, evaluating the advantages of triclosan-coated compared with non-coated sutures in preventing SSIs for fascia closure of laparotomy in abdominal gastrointestinal surgery. To identify prospective randomized trials regarding this topic, we searched Cochrane Central Register of Controlled Trials (Central) and PubMed with the following search terms: “triclosan or triclosan coated;” “surgical site infection;” and “randomized controlled trial” was searched, respectively. To avoid the evaluation of the heterogenous group of patients, the following studies were excluded; only emergency surgery, or not including gastroenterological surgery. The Mantel-Haenszel random-effects model was performed with R software (CRAN, *R*3·6·2; https://cran.r-project.org/).

**Results:**

This meta-analysis included eleven phase-III and two prospective studies, which comprised 9588 patients. The aggregated phase-III results of the trials demonstrate a significant superiority of triclosan-coated sutures compared with non-coated sutures (random-effect model, OR 0.71, 95 % CI 0.56–0.90, *P* = 0.0052).

**Conclusion:**

The meta-analysis showed benefit with triclosan-coated sutures in preventing SSI after gastrointestinal surgery.

## Introduction

Surgical site infection (SSI) is one of the most common postoperative complications after abdominal surgery. Well-known risk factors related to SSI include patients' age, comorbidities, such as drinking habit, smoking, obesity, diabetes, hypertension, steroid intake, and immunosuppression. In preventing SSI, advances have been made such as antibiotic prophylaxis, skin disinfection, mechanical bowel preparation, and hand decontamination. The recent approach is the use of triclosan-coated sutures for the abdominal fascial closure. These products are available in the market for use as triclosan-coated polydioxanone antimicrobial sutures (PDS Plus; Ethicon Inc). As the National Institute for Health and Care Excellence (NICE) guidelines stated, antimicrobial-coated sutures have the effectiveness of preventing SSIs limited to particular types of surgery. The recommendation is derived from the results of several situations such as different patients, surgical procedures, wound classes, suture materials. In this systematic review, we conducted a meta-analysis to analyze the efficacy of the antimicrobial-coated sutures compared to uncoated sutures for prevention of SSIs. Patients had undergone abdominal surgery including gastrointestinal surgery with wound classifications ≥2 (clean-contaminated).

### Meta-analysis

We did a meta-analysis, including present outcomes to evaluate the advantages of triclosan-coated compared with non-coated sutures in preventing SSI for fascia closure of laparotomy in abdominal surgery. To identify the prospective randomized trials, we searched Cochrane Central Register of Controlled Trials (Central) and PubMed with the following search terms: “triclosan or triclosan coated;” “surgical site infection;” and “randomized controlled trial” was searched. To avoid the evaluation of the heterogenous group of patients, the following studies were excluded; only emergency surgery, or not including abdominal, gastrointestinal surgery. All titles and abstracts retrieved were reviewed by independently by two investigators at Osaka University and Monash University. We performed the meta-analysis with the Mantel-Haenszel random-effects model, using R software (CRAN, *R*3·6·2; https://cran.r-project.org/). This systematic evaluation was not registered to public database. The statistical methods of this study were reviewed by Miyoshi N.

## Results

**Fig. 1:**
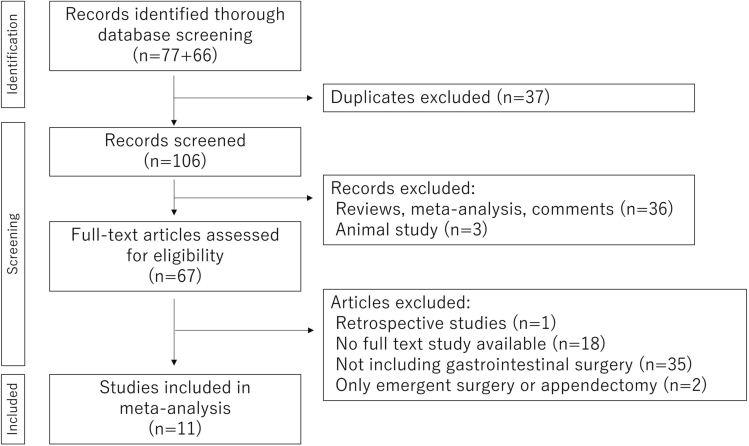
Flow diagram for study selection. Flow diagram shows the identification of studies searched by databases.

This meta-analysis included eleven phase-III and two prospective studies, which comprised 9588 patients [[1], [2], [3], [4], [5], [6], [7]]. (Fig. 1). Four of these studies assessed triclosan-coated polyglactin 910 braided suture material (Coated Vicryl Plus, Ethicon Inc) compared with non-coated polyglactin 910 (Vicryl; Ethicon) [1,2,5]. Four studies compared PDS Plus and PDS II [1,7]. Five trials were performed in multi-center settings [1,2,4]. Four focused on colorectal surgery only [1,2], whereas seven trials evaluated a mixed cohort of patients who underwent general and abdominal surgery [1,[3], [4], [5], [6], [7]]. Eight trials used the SSI definition of the Centers for Disease Control and Prevention [[1], [2], [3], [4], [5], [6]]. The funding source was described specifically in three studies [1,4,6]. We can evaluate clean-contaminated stratum in three studies including FALCON study to avoid the heterogeneity of the population including contaminated or dirty stratum, similar to the population of elective surgery. Six phase-III trials showed benefit significantly in preventing SSI in the triclosan-coated group [1,[5], [6], [7]], whereas five trials did not show significant differences between the treatment groups [[1], [2], [3], [4]].

**Fig. 2:**
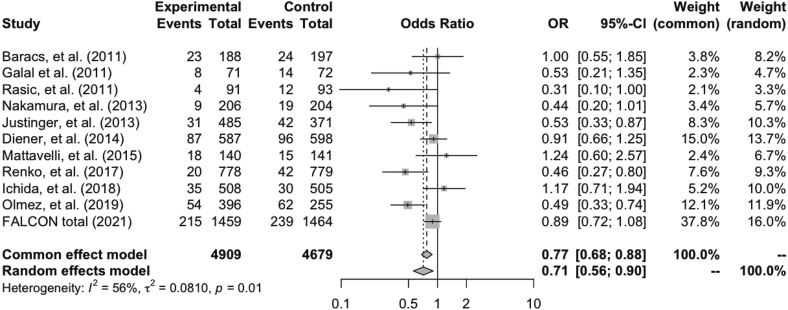
Meta-analysis of the prospective randomized trials. Meta-analysis of the prospective randomized trials compares the efficacy of using triclosan-coated and uncoated sutures for abdominal fascia closure after midline laparotomy, in preventing surgical site infections. Abbreviations: OR, odds ratio; CI, confidence interval.

**Fig. 3:**
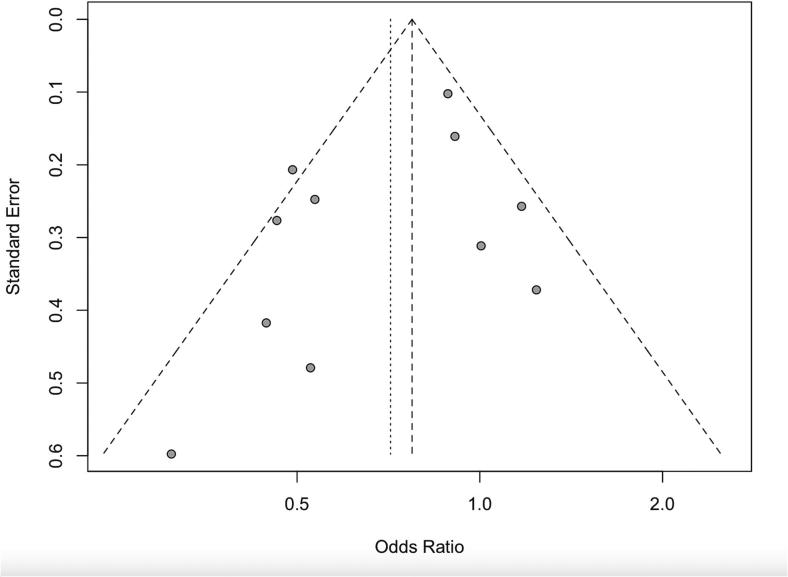
Funnel plots based on the suture materials preventing surgical site infection. Studied evaluate the advantages of triclosan-coated compared with non-coated sutures in preventing SSI for fascia closure of laparotomy in abdominal surgery.

The aggregated phase-III results show significant benefit with triclosan-coated sutures in preventing SSI, compared with non-coated sutures (random-effect model, OR 0.71, 95 % CI 0.56–0.90, *P* = 0.0052) (Fig. 2). Tau-squared presenting the between study vias is 0.081. The funnel plot used to investigate the publication bias is shown in Fig. 3.

## Discussion

Our meta-analysis shows that using triclosan-coated PDS/Vicryl Plus sutures for fascia closure reduces the rate of SSI in abdominal gastrointestinal surgery, evaluating eleven phase-III trials comprising 9588 patients. As the limitations of our meta-analysis, the patients background was similar but different in studies. Seven trials evaluate a mixed cohort of patients who underwent general and abdominal surgery. And three studies evaluating clean-contaminated stratum to avoid the heterogeneity of the population which includes contaminated or dirty stratum, because they are similar to the population of elective gastrointestinal surgery. As the suture type, braid or monofilament, was not analyzed separately. However, our study can provide answers whether coated or uncoated sutures are better for reducing SSI in elective gastrointestinal surgery.

## Conclusion

In our meta-analysis evaluating eleven phase-III trials comprising 9588 patients, the results clearly demonstrate significant benefit with triclosan-coated sutures over non-coated suture material.

## CRediT authorship contribution statement

NM conceived and designed the study, prepared and wrote the report, and participated in data analysis. SM designed the study and participated in data acquisition. NM and SF contributed to data interpretation, proof reading and writing. CSGOCG contributed to the data interpretation.

## Funding sources

This research did not receive any specific grants from funding agencies in the public, commercial, or not-for-profit sectors.

## Ethical approval

The multicenter protocol of this trial was approved by the ethics committee of the Osaka University, Japan (Reference number: 16250-6).

## Declaration of competing interest

The authors have no conflicts of interest to disclose.

## Data Availability

All data analyzed in this study are included in this published article.
